# Diallelic Analysis of Tropical Maize Germplasm Response to Spontaneous Chromosomal Doubling

**DOI:** 10.3390/plants9091224

**Published:** 2020-09-17

**Authors:** Vijay Chaikam, Manje Gowda, Leocadio Martinez, Gregório Alvarado Beltrán, Xuecai Zhang, Boddupalli M. Prasanna

**Affiliations:** 1 International Maize and Wheat Improvement Center (CIMMYT), ICRAF Campus, UN Avenue, Gigiri, P.O. Box 1041–00621, Nairobi, Kenya; V.Chaikam@cgiar.org (V.C.); M.Gowda@cgiar.org (M.G.); 2 International Maize and Wheat Improvement Center (CIMMYT), Apdo, Postal 6-641, Mexico 06600, Mexico; L.Martinez@cgiar.org (L.M.); g.alvarado@cgiar.org (G.A.B.); XC.Zhang@cgiar.org (X.Z.)

**Keywords:** maize, doubled haploid, haploid male fertility, haploid fertility, diallel analysis, genomic prediction

## Abstract

Chromosome doubling is an important step in the production of maize doubled haploid (DH) lines to induce fertility in the male and female reproductive organs of haploid plants. Chromosomal doubling is routinely accomplished by treating haploid seedlings with mitosis-inhibiting chemicals. However, chromosomal doubling involves several labor-intensive steps and toxic chemicals. Spontaneous chromosomal doubling without any chemical treatments occurs at high frequency in haploids from a few maize genotypes. This study focused on elucidating the genetic components of two traits important for using spontaneous doubling in maize-breeding programs, namely, haploid male fertility (HMF) and haploid fertility (HF). In two different sets of diallel crosses, haploids were derived and assessed for HMF and HF in two environments in replicated trials. The results revealed significant genotypic variations for both traits. The general combining ability (GCA) and specific combining (SCA) were significant for both traits. Significant and positive GCA effects of up to 14% and 9% were found for HMF and HF, respectively. No significant reciprocal effects and genotype-by-environment (G×E) interactions were found for HF in both experiments, but HMF showed significant effects for both in one of the experiments. The GCA effects were more important than the SCA effects for HMF and HF across environments, implying that selection could facilitate their improvement. The high correlations between F1-hybrid performance and mid-parent values, as well as that between F1-hybrid performance and GCA effects, also supports the assumption that these traits are controlled by a few genes. SCA effects also played a role, especially when lines with low spontaneous doubling were used as parents. Overall, spontaneous doubling can be introgressed and improved in elite germplasm with selection, and it has the potential to be employed in DH pipelines.

## 1. Introduction

The technology for generating plants with a gametic number of chromosomes and using them to produce completely homozygous inbred lines is generally referred to as doubled haploid (DH) technology. DH technology can fix gametes from segregating populations into homozygous inbred lines in a single generation, thereby dramatically accelerating the pace at which improved cultivars can be developed. DH technology is recognized as one of the important tools in maize breeding to accelerate genetic gains. The use of DH lines in maize breeding increases the genetic variance among the lines tested, phenotype-to-genotype correlation and selection accuracy [1,2]. Together, DH greatly improves the efficiency of breeding programs and potentially reduces the costs in the development of improved cultivars.

DH technology in maize encompasses (1) the production of haploids by crossing the germplasm from which the DH lines are anticipated with pollen from maternal haploid inducers, (2) the identification of haploids from diploids at the seed or seedling stage using genetic markers or natural differences among haploids and diploids, (3) subjecting the haploid seedlings to chromosomal-doubling treatments using mitosis-inhibiting chemicals, and (4) caring for the treated haploid plants in the field (referred to as D_0_ nursery) and self-pollinating fertile DH plants to produce seed for DH lines (D_1_ lines). The process of maize DH-line production is well documented in several publications [2,3,4]. Great advances have been made in improving the efficiencies of haploid induction and identification [2]. However, in the step of chromosomal doubling and the production of seed for DH lines, there is considerable scope for improving the efficiency and reducing the costs. The chromosomal-doubling step encompasses growing haploid seedlings in a growth chamber or a greenhouse, treating them with mitosis-inhibiting chemicals, post-treatment recovery in the greenhouse, and transplanting the seedlings to the field with some protocol-specific modifications [2,4]. The chemicals most commonly used for chromosomal doubling are colchicine, antimitotic herbicides, and N_2_O gas [5,6,7]. The purpose of chromosomal-doubling treatments is to produce pollen and form seeds from reproductively sterile haploid plants [8]. Upon chromosomal-doubling treatments, some proportion of surviving haploids may double the chromosomes in the reproductive tissues and produce pollen and seed. The overall success rate (OSR) is a criterion that measures success for artificial chromosomal-doubling methods [7], and the OSR varies from 5 to 35% depending on the protocol and the chemical used [6,7,9]. Even though artificial chromosome-doubling methods are extensively practiced in the DH-production pipelines, this process demands considerable manual labor and laboratory and greenhouse facilities, besides employing toxic chemicals [2,10]. Hence, breeding programs are seeking alternative methods that can be operationally simpler, less expensive and not involve toxic chemicals.

A high proportion of haploids from very few maize genotypes show fertility without the application of anti-mitotic chemicals through spontaneously doubling their chromosomes, even though the reasons are not understood well [11]. The genotype dependency of spontaneous doubling has limited its widespread use in DH pipelines to date. If this limitation can be addressed, spontaneous chromosomal doubling could dramatically simplify the chromosomal-doubling step and reduce the costs significantly [10]; hence, it is pursued as an alternative to artificial chromosomal-doubling methods. Most haploid plants are infertile in their male reproductive organs, namely, tassels, but female reproductive organs, namely, ears, produce seed when pollinated with pollen from diploid plants even without being subjected to artificial chromosomal-doubling treatments [12,13]. Hence, the lack of haploid male fertility (HMF) is a constraint for producing seed for DH lines when relying on spontaneous doubling. The analysis of HMF in large numbers of inbreds showed greater genetic variance in temperate [14] and tropical germplasm [11]. QTL-mapping studies using bi-parental populations [15,16,17,18] and genome-wide association studies (GWAS) [11,14] indicated that HMF is controlled by a few QTLs with major effects. Another major aspect of spontaneous doubling that is also important for DH-production pipelines is overall haploid fertility (HF) [11] or overall fertile haploid plants [19], which considers both male and female fertility and is based on seed production from DH plants. A greater genetic variance was also detected for HF in tropical inbred lines, and GWAS identified 11 significantly associated single-nucleotide polymorphisms (SNPs) with HF [11]. However, HF is less studied than HMF, and there are no previous studies revealing the genetic components and inheritance of this important trait.

In maize-breeding programs, combining-ability analyses are widely used to generate genetic information, such as general combining ability (GCA) and specific combining ability (SCA), for use in genetic-diversity evaluation, parental selection and hybrid development [20,21,22]. Combining-ability analyses are always performed through diallel mating methods developed by Griffing [23]. A diallel analysis of HMF involving six temperate inbreds revealed significant GCA and SCA effects [24]. Additive effects were noted to be important for the selection of HMF, while epistatic effects could also play a role [25]. The HMF can be increased by single-plant selection and recurrent selection [25]. However, such information is not available for tropical germplasm. Moreover, there are no studies reported to date on the GCA and SCA effects for HF. Therefore, this study was designed to (i) evaluate the GCA and SCA effects for HMF and HF, and the maternal effects of selected maize inbred lines, and (ii) predict the performance of the hybrid for HMF and HF based on mid-parent value and GCA effects.

## 2. Results

### 2.1. Genetic Distance among the Inbred Lines

**Figure 1:**
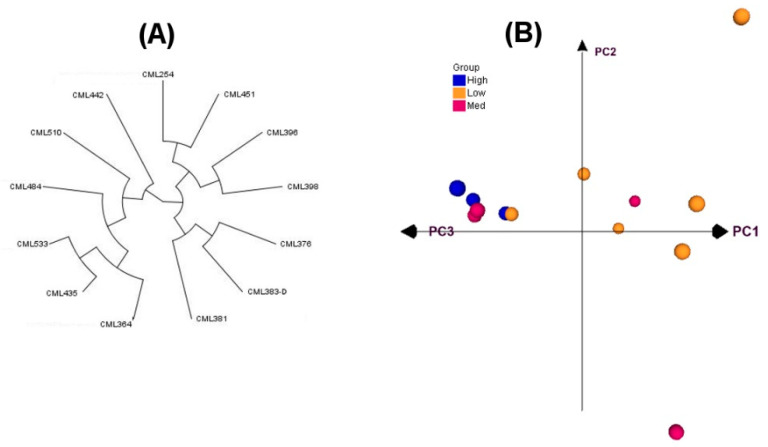
Dendrogram (**A**) and principal component analyses (**B**) of 13 (CMLs) based on modified Rogers’ distances estimated using 10,000 Genotyping by Sequencing (GBS) markers.

Rogers’ genetic distance estimates based on 190,000 Genotyping by Sequencing (GBS) SNP marker data revealed a high variation in the relatedness among the parental lines. The genetic distances among the 13 parental lines used in both diallel experiments ranged from 0.35 to 0.44, with a mean of 0.42. In the 78 pairwise comparisons, only 11.5% had genetic distances <0.40. Most of the pairs of parents (87.5%) fell between 0.40 and 0.44 in distance. The principal component analyses (PCAs) showed clustering among the inbred lines (Figure 1). The first two principal components (PCs) explained 17% of the total variation. The lowland tropical inbreds developed in Columbia and Mexico formed two separate and distinct clusters. Similarly, subtropical/midaltitude International Maize and Wheat Improvement Center (CIMMYT) maize lines (CMLs) from Mexico (except CML484) and Zimbabwe formed separate clusters. CML484 showed more closeness to lowland tropical inbreds from Columbia.

### 2.2. Phenotypic Variation for HMF and HF in the Lines and Hybrids

A significant variation for both HMF and HF was found among the parental lines used in both the diallel experiments (Table 1). In the first diallel experiment (Diallel I), CML364, CML435 and CML442 exhibited very high HMF and HF exceeding 65% and 25%, respectively. CML376 and CML484 showed medium levels of HMF (~30%) and HF (~11%). CML533, CML383 and CML254 showed very low HMF and HF, at less than 5%. In the second diallel experiment (Diallel II), only CML364 showed very high HMF and HF. CML396 and CML510 showed medium levels of HMF and HF. The other four inbreds (CML381, CML383, CML451 and CML533) showed low levels of HMF and HF.

**Figure 2:**
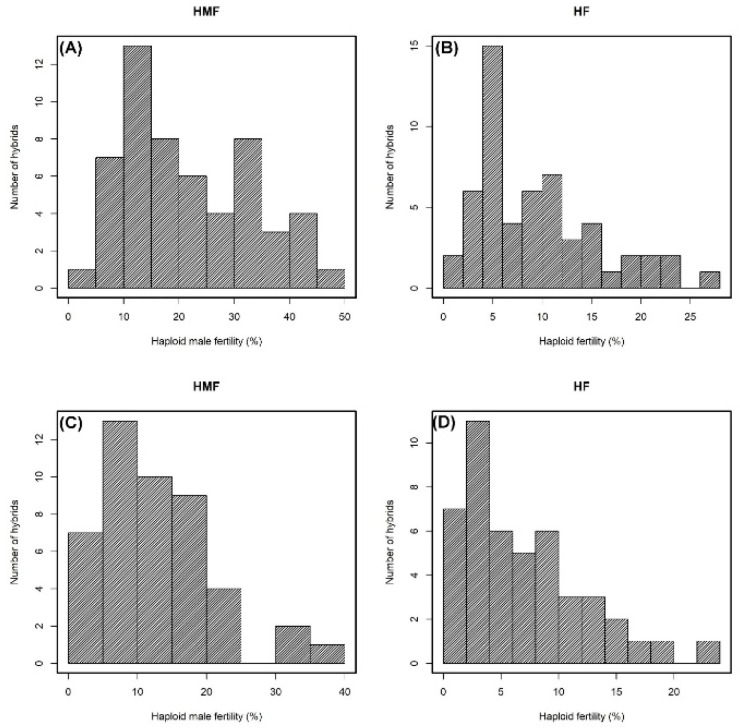
Phenotypic distribution of F1 hybrids for HMF and HF in Diallel I (**A**,**B**) and II (**C**,**D**) experiments.

Among the hybrids, wide variations for both HMF and HF were noticed in both the diallel experiments (Figure 2 and Table 2). The mean performance of the hybrids for HMF and HF was 23.91% and 10.67%, respectively, in Diallel I. The mean performance of hybrids for HMF and HF in Diallel II was 12.59% and 6.9%, respectively. For HMF, the mean performance ranged from 2.36 to 46.83% in Diallel I and from 2 to 37.61% in Diallel II. For HF, the mean ranged from 1.58 to 27.59% in Diallel I and from 0.36 to 23.83% in Diallel II. The performance of the hybrids (Table 2) was low compared to that of the inbreds (Table 1) for both HMF and HF. Among the different hybrid combinations, the HMF rate was the highest for the high HMF × high HMF and high HMF × medium HMF hybrid combinations in both the experiments. HF was also the highest among the high HF × high HF and high HF × medium HF hybrid combinations in both experiments. HMF and HF were the lowest in the low × low and some medium × low hybrid combinations in both experiments. Medium levels of HMF and HF were found in hybrids involving parents with medium and low HMF and low HF.

Analyses of variance (ANOVAs) for HMF and HF for both of the diallel experiments are shown in Table 3. The mean squares for HMF and HF were statistically significant for the hybrids in both diallel experiments, suggesting that variation among the hybrids existed for both the traits. Based on Griffing’s Method 1, the genotype or hybrid variances of these traits were further partitioned into GCA and SCA, and reciprocal variances, into maternal and non-maternal variances. The mean squares were significant for both GCA and SCA in both diallel experiments for both HMF and HF. The mean squares for reciprocal effects and maternal effects were significant only for HMF in the Diallel II experiment. The mean squares for reciprocal effects and maternal effects for HF were not significant in either diallel experiment. The environment effect was only significant for HMF in the Diallel I experiment. The environment x hybrid interaction was significant for HMF in the Diallel II experiment. Environment interactions with SCA, reciprocal crosses and maternal effects were significant for HMF in the Diallel II experiment but not in the Diallel I experiment. Interactions between environment and SCA, reciprocal crosses and maternal effects were not significant for HF in either diallel experiment.

Estimates of the additive variance, dominance variance and heritability are presented in Table 4, along with the GCA/SCA ratio as Baker’s ratio. The additive genetic variance (σ^2^_A_) was higher than the dominance genetic variance (σ^2^_D_) in both the experiments for both HMF and HF. The Baker’s ratios for HMF and HF were 0.73 and 0.70 in the Diallel I experiment and 0.78 and 0.71 in the Diallel II experiment, respectively. A moderate magnitude of the narrow-sense heritability was observed compared to that of the broad-sense heritability.

### 2.3. General- and Specific-Combining-Ability Analyses

Estimations of GCA effects for HMF and HF for both diallel experiments are presented in Table 5. In the first diallel experiment, the GCA effects were positive and significant for HMF for all the three inbreds, which had high rates of spontaneous doubling (CML364, CML435 and CML442). The GCA effects were positive and significant for HF for CML364 and CML435, while they were positive but not significant for CML442. For all the three inbreds with low spontaneous doubling (CML533, CML383 and CML254), the GCA effects were significant but negative for both HMF and HF. Among the two inbreds with medium levels of spontaneous doubling (CML483 and CML376), CML483 showed positive but nonsignificant GCA effects, while CML376 showed a negative, nonsignificant GCA effect for HMF and a negative significant effect for HF. CML364 produced the highest positive GCA effect for HMF (13.01%) and HF (8.28%). CML533 produced the highest negative GCA effect for both HMF (−12.05%) and HF (−6.40%).

In the second diallel experiment, CML364, with high spontaneous doubling, showed positive and significant GCA effects (Table 5). The two inbreds with medium spontaneous doubling rates (CML396 and CML510) showed positive and significant GCA effects for HMF. For HF, CML396 showed positive and significant effects, while CML510 showed positive but nonsignificant GCA effects. Among the five inbreds with low spontaneous doubling (CML381, CML383, CML398, CML451 and CML533), all inbreds except CML451 showed negative and significant GCA effects. CML364 showed the highest positive GCA effect for both HMF (14.36%) and HF (8.61). CML383 and CML533 both showed similarly low levels of GCA effects for both HMF and HF.

**Figure 3:**
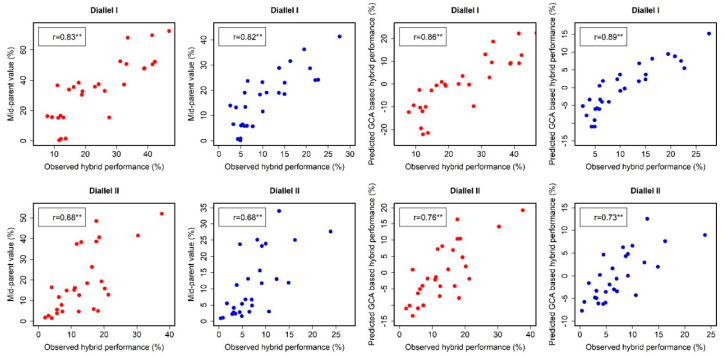
Prediction of hybrid performance for HMF (red dots) and HF (blue dots) based on means of the parental value and GCA effects in two diallel experiments.

The correlations between the mid-parent value and F1-hybrid performance were high for both HMF and HF (Figure 3). In the first diallel experiment, the correlations between the mid-parent value and F1-hybrid performance for HMF and HF were 0.83 and 0.68, respectively. In the second diallel experiment, the correlations between the mid-parent value and F1-hybrid performance for HMF and HF were 0.82 and 0.68, respectively. The correlation between the F1 performance and GCA effects for HMF and HF were 0.86 and 0.89 in Diallel I, and 0.76 and 0.73 in Diallel II, respectively.

Estimates of the SCA effects for HMF and HF for the two diallel experiments are shown in Table 6. Of the 28 hybrids in the first diallel experiment (excluding reciprocals), the SCA effects for HMF were significant only for six hybrids, where the SCA effects for HMF were only positive for two hybrids, CML254 × CML533 and CML383 × CML533. For HF, all the significant SCA effects were negative. Among the 28 hybrids in the second diallel experiment, the SCA effects for HMF were significant for ten hybrids, where only three hybrids—CML381 × CML396, CML381 × CML398 and CML398 × CML533—showed positive SCA effects for HF (Table 6). For HF, the SCA effects were significant for four hybrids in the second diallel experiment, where only one hybrid, CML381 × CML396, showed a positive SCA effect.

## 3. Discussion

Improving the chromosomal-doubling efficiency in maize haploids can unfold the full potential of DH technology, which could facilitate new selection approaches that are currently not feasible due to the lower efficiency of artificial chromosomal-doubling methods. High chromosomal-doubling efficiency can facilitate phenotypic or marker-assisted selection for simple traits and genomic selection for complex traits at the haploid stage [10]. This could reduce breeding-cycle time and increase selection intensity and efficiency. This study confirmed previous observations [11,14] that the spontaneous chromosomal-doubling efficiency can be very high in a few maize genotypes compared to the doubling efficiency achieved by artificial chromosomal-doubling protocols. The use of spontaneous doubling can substantially reduce the labor costs, and accordingly, overall costs in DH-line production may be reduced by 20–50% [10,25]. However, as a high level of spontaneous doubling is limited to a few genotypes, breeding strategies need to be developed for using spontaneous doubling in DH-production pipelines. For this, estimating variance components and combining ability is important, and this is the first study reporting such estimates in the tropical inbred lines.

Two traits associated with spontaneous chromosomal doubling, namely, HMF and HF, showed greater variation in the inbred lines used in this study, and the data agree with a previous report on these lines [11]. The significant genotype (hybrid) effect observed in ANOVA indicated considerable variability for HMF and HF among the F1 hybrids evaluated in this study. Observing significant GCA and SCA variances suggests that the performance of hybrids can be attributed simultaneously to both additive and non-additive genetic components. The environment had no significant effect on HF in both diallel experiments, while it strongly affected the HMF in the first diallel experiment. Fuente et al. [24] also observed a significant environment effect for HMF. In both diallel experiments, non-significant GCA x environment-interaction variances were observed for both HMF and HF, indicating that the parental inbred lines do not perform differently in different environments. The significant SCA × environment-interaction variance for HMF observed in the second diallel experiment indicates the importance of non-additive genetic effects in this set of F1 crosses. The non-significant reciprocal variance and maternal variance for HF indicates that extra-nuclear factors are not important in the inheritance of HF. However, they seemed to play a role for HMF in the second diallel experiment.

The larger proportion of the mean squares of the GCA compared to SCA for HMF and HF suggests that the additive genetic effect was the main contributor to their inheritance in the F1 hybrids in the two evaluated environments. In a diallel experiment with a fixed-effects model, even though the estimates of additive and dominance variances are less important [26], the GCA–SCA ratio (GSR) proposed by Baker [27] (Baker’s ratio) is frequently used in several studies [28,29,30] to determine the relative importance of GCA and SCA. If the Baker’s ratio is close to 1, then GCA is predominant for a given trait. The Baker’s ratios >0.7 for both the traits in both diallel experiments also indicated that GCA was relatively more important than SCA for both the traits. In addition, the higher additive genetic variance (σ^2^_A_) compared to dominance variance (σ^2^_D_) in both the experiments and higher estimates for the narrow-sense heritability over those for the broad-sense heritability also indicate the predominance of the additive effects for both HMF and HF in tropical maize.

All the F1 hybrids (excluding reciprocals) assessed in the two diallel experiments revealed a varied magnitude of SCA effects in different groups (Table 6). In a group of hybrids derived from parents with a high rate of HMF, significant SCA effects were observed in a negative direction. This is probably one of the reasons for the low performance of hybrids derived from high x high HMF combinations, compared to their parents’ per se performance. Other possible reasons may be that HMF and HF are known to be controlled by a few genes with large effects, and these genes might have been fixed in the selected lines, which had a high performance of >65% for HMF and >26% for HF. Fuente et al. [24] also observed that the F1 diallelic crosses show significantly less HMF compared to inbreds with high HMF in temperate maize germplasm. Contrary to the hybrids developed from lines with high and medium levels of HMF and HF, the hybrids developed with poor HMF- and HF-performing lines exhibited significant but positive SCA effects. Thus, non-additive effects play an important role in lines with poor HMF and HF performance. Non-additive effects generally include both dominance and epistatic effects. For the HMF and HF measured in haploid plants, the SCA effect might be partially determined by additive x additive epistatic effects only, as dominant effects are not possible due to a single copy of chromosomes in haploids [24]. Even if the genome is doubled spontaneously at a very early stage, the duplicated chromosomes will be exact replicas of existing chromosomes, eliminating the possibility of dominant effects [24]. Improving spontaneous doubling should be based on breeding strategies that exploit additive effects. The inbred lines CML364, CML435 and CML442 had the highest significant positive GCA effects for both HMF and HF, suggesting that these lines may harbor loci conferring a large positive effect and transmit such favorable alleles to their progenies through hybridization. These lines should also be useful as sources of QTLs for marker-assisted selection that would facilitate the transfer of alleles favorable for increased spontaneous doubling into commercial elite lines and hybrids.

The correlation between the mean parental values and F1-hybrid performance for HMF and HF in both the diallel experiments clearly indicates that merely knowing the per se performance is enough for predicting the performance of hybrids for HMF and HF with limited field experiments. Furthermore, by knowing the GCA effects of lines, the accuracy for finding the best possible combinations with high HMF and HF is also high (Figure 3). These high correlations indicate the presence of a few genes controlling most of the variation of these two traits, as indicated by several genetic studies [11,14,15,16,17,18], and fixing these genes in lines could lead to high rates of spontaneous doubling. Focusing on a few genes for increasing spontaneous doubling also encourages the use of marker-assisted backcrossing to introgress these genes into other elite lines. The possibility of eliminating or reducing chemical-based chromosomal doubling in the process of DH-line development could ultimately save resources for maize-breeding programs.

## 4. Materials and Methods

### 4.1. Plant Materials

In our previous study, 400 CIMMYT maize lines (CMLs) were characterized for their response to spontaneous chromosomal doubling, which indicated a wider variation for HMF and HF in tropical inbred lines [11]. In this study, a total of thirteen inbred lines were chosen to make two separate diallelic crosses. The seeds for these inbred lines were obtained from the CIMMYT maize germplasm bank. Each diallel experiment was performed with eight CMLs; the CML364, CML383 and CML533 were common to both diallel experiments. Among the thirteen inbreds, seven inbreds show adaptation to lowland tropics, and six inbreds, to subtropical/midaltitude regions. Among the seven CMLs adapted to lowland tropics, three were developed in Columbia and four were developed in Mexico by CIMMYT maize-breeding programs. Among the six subtropical-/midaltitude-adapted inbreds, four were developed in Mexico and two were developed in Zimbabwe by CIMMYT maize-breeding programs. The first diallel experiment included three CMLs with high spontaneous-doubling capability, two CMLs with medium spontaneous-doubling capability and three CMLs with low spontaneous-doubling capability. The second diallel included one CML with high spontaneous-doubling capability, two CMLs with medium spontaneous-doubling capability and four CMLs with low spontaneous-doubling capability. Full sets of 64 F1 crosses including reciprocal crosses were made from each set of diallels in the winter cycle of 2018 at the CIMMYT experimental station at Agua Fria (20.26° N, 97.38° W) in Mexico.

### 4.2. Haploid Induction and Identification

The diallelic populations and parental inbred lines were grown in four rows and crossed to the tropical haploid-inducer hybrid of CIM2GTAIL1009 × CIM2GTAIL006 [31] in the 2018 summer cycle at CIMMYT’s Agua Fria experimental station in Mexico to induce haploids. The haploid seeds were separated from diploid seeds based on *R1-nj*-marker expression [32]. In Diallel 1, inbreds CML376, CML435 and CML533 showed complete inhibition of *R1-nj*-marker expression [33]. The hybrids formed between any two of these inbreds also showed complete inhibition of the *R1-nj* marker. Hence, haploids were identified based on their strikingly poor vigor, erect leaves and paleness of leaves, as described earlier [34]. In the hybrids using CML376, CML435 or CML533 as one of the parental lines, and in which partial inhibition was noticed, the required number of haploids could be identified based on *R1-nj*-marker expression.

### 4.3. Field Trials and Trait Assessment

For each diallelic cross and parental line, 100 putative haploid kernels were planted in Mexico, directly in the field in the summer season of 2019 at Metztitlan (20.6° N, 98.76° W) and winter season of 2020 at Agua Fria using an alpha-lattice design with two replications. For the hybrids with complete inhibition of *R1-nj*-marker expression, haploids were identified in the lab based on the seedling traits and transplanted to the field. Any false positives observed were eliminated based on their superior plant vigor and broad and drooping leaves after 20–30 days of planting [11,34]. Haploids with tassel fertility were selfed two to three times on consecutive days depending on the pollen availability. Data on the number of haploid plants surviving at anthesis, the number of haploid plants with tassel fertility and the number of haploid plants with seed were recorded and used to calculate the HMF and HF, as described earlier [11]. The HMF was calculated as a proportion of the total number of pollen-producing haploid plants from the total number of haploid plants per plot and expressed as a percentage. The HF was calculated by dividing the total number of seed-producing haploids by the total number of haploid plants per plot and expressed as a percentage.

DNA from all 13 inbred lines was extracted from seedlings having 3–4 leaves and genotyped using a Genotyping by Sequencing (GBS) platform at the Institute for Genomic Diversity, Cornell University, Ithaca, USA, as per the procedure described in earlier studies [11,35]. The GBS SNP datasets were filtered, where a minor allele frequency <0.10, heterozygosity f >5% and missing data rates >5% were exclusion criteria for further analysis in TASSEL ver 5.2 [36]. Finally, we used 10,000 SNPs for further analyses. Principal coordinate analyses (PCoAs) were performed and dendrograms created with TASSEL ver 5.2 and then visualized in the R software (http://www.R-project.org/).

## 5. Data Analysis

The statistical analysis was carried out using the AGD-R (Analysis of Genetic Designs with R for Windows) Version 5.0 statistical software [37]. Analysis of variance (ANOVA) was performed using the following statistical model:

*y_ijkm_* = *µ + g_ij_* + *(gl)_ijk_* + *r_mk_* + *e_ijkm_* (1)

where *y_ijkm_* represents the phenotypic performance of the *ij*th genotype (parental line *i* = *j*, or hybrid *i*≠*j*) in the *m*th replication in the *k*th environment, *µ* is an intercept term, *g_ij_* is the genetic effect of the *ij*th genotype (parental line *i* = *j*, or hybrid *i*≠*j*), *l_k_* is the effect of the kth environment, *(gl)_ijk_* is the interaction of the *ij*th genotype (parental line *i* = *j*, or hybrid *i*≠*j*) with the *k*th environment, *r_mk_* is the effect of the *m*th replication in the *k*th environment, and *e_ijkm_* is the residual error term.

The diallel analysis was carried out by applying Griffing’s Model I (fixed model) Method 1 (parents and hybrids with reciprocal crosses) [23]. The mean squares for GCA and SCA were tested against their respective error variances inferred from ANOVA using the SAS software program version 9.4 [38]. The variance was partitioned into GCA, SCA, reciprocal, maternal and nonmaternal components and their interactions with years. To test the mean squares for the GCA, SCA, reciprocal, maternal and nonmaternal components for significance in the two-location analysis, the interaction between locations and the corresponding component was used as the error term for *F* tests. Estimates of the GCA and SCA effects were calculated, and their significance was determined by *t* tests. Further variance-components estimates were computed for GCA, SCA and reciprocal (REC). All the calculations were performed using the AGD-R software. Pairwise Pearson’s correlation coefficients (r) were calculated between the F1 hybrids (F1P) and mid-parent values (MPV), r(MPV: F1P). Furthermore, we also tested the Pearson’s correlation of F1P with the sum of the GCA effects of both the parents, r(GCA: F1P), for each diallel experiment.

## 6. Summary

The significant GCA effects compared to the SCA effects of spontaneous-doubling-associated traits, namely, HMF and HF, indicate that breeding programs can improve these traits based on additive genetic effects. It is possible to predict the hybrid performance for HMF and HF based on the per se performance of inbred lines. Information on the GCA effects of the parental lines could be used to improve the prediction and forecast the best possible hybrid combinations for both the traits. Three tropical inbred lines with high GCA effects for both the traits were identified, which can be used to transfer the high spontaneous-doubling response to elite tropical maize germplasm through either recurrent selection or marker-assisted selection. Breeding programs, especially with resource limitations, could greatly benefit from such improved germplasm, as the need for artificial chromosome doubling is eliminated, which needs laboratory and greenhouse facilities.

## Figures and Tables

**Table 1:** Haploid male fertility (HMF) and haploid fertility (HF) of maize lines used in this study expressed in %. The pedigrees, adaptation and country where the lines were developed are also indicated.

Line	Pedigree	Adaptation	Country	HMF	HF
Diallel I					
CML364	SAHC1-5-1-1-5-3-B	LLT	Colombia	74.33	46.05
CML435	SA3-C4HC(16x25)-2-4-3-6-B-B-B-B-B	LLT	Colombia	65.30	36.69
CML442	[M37W/ZM607#bF37sr-2-3sr-6-2-X]-8-2-X-1-BBB	ST/MA	Zimbabwe	70.90	26.54
CML376	SLWHG-AF118-2-1-1-B-1-B-1-B-B	ST/MA	Mexico	30.23	11.76
CML484	G 19 C3H104-3-1-B-2-3-BB	ST/MA	Mexico	30.75	11.51
CML533	SA4C4HC19-3-2-2-2-3-4-3-B-B-B-B	LLT	Colombia	2.44	1.43
CML383	P502c1#-771-2-2-1-1-B	ST/MA	Mexico	0.62	0.41
CML254	TUXSEQ-149-2-BBB-##-1-BB-f	LLT	Mexico	0.15	0.00
Diallel II					
CML364	SAHC1-5-1-1-5-3-B	LLT	Colombia	74.33	46.05
CML396	P21C5HC109-3-1-5-4-B-4-3-##-2-B*6	LLT	Mexico	22.82	21.99
CML510	SW89300-1P5S2-5-##1-6-BB	MA	Zimbabwe	29.87	9.31
CML398	P21 C5HC216-2-3-B-#*4-BBB-###-B*8	LLT	Mexico	7.01	4.24
CML451	[NPH28-1*G25)*NPH28]-1-2-1-1-3-1-B*6	LLT	Mexico	8.85	4.09
CML381	P501c1#-401-3-1-2-B-B	ST/MA	Mexico	3.00	1.78
CML533	SA4C4HC19-3-2-2-2-3-4-3-B-B-B-B	LLT	Colombia	2.44	1.43
CML383	P502c1#-771-2-2-1-1-B	ST	Mexico	0.62	0.41

LLT = Lowland tropics; ST/MA: Subtropics/midaltitude; HMF = Haploid male fertility; HF: Haploid fertility.

**Table 2:** Mean performance of hybrids across two environments for HMF and HF in Diallels I and II.

Diallel I	BLUEs (%)		Diallel II	BLUEs (%)	
Hybrids	HMF	HF	Hybrids	HMF	HF
Group1: High × High			Group 1: High × High		
CML364 × CML442	46.83	19.47	CML364 × CML510	37.61	23.83
CML435 × CML364	41.40	27.59	Group 2: High × Medium		
CML435 × CML442	33.56	16.31	CML364 × CML396	17.61	12.82
Mean	40.60	21.12	Group 3: High × Low		
Group 2: High × Medium			CML381 × CML364	17.62	9.98
CML364 × CML376	42.23	13.74	CML383 × CML364	11.64	9.14
CML442 × CML376	41.51	10.86	CML398 × CML364	18.52	8.18
CML435 × CML484	38.93	22.03	CML451 × CML364	30.32	16.22
CML435 × CML376	38.78	22.69	CML533 × CML364	13.03	4.47
CML442 × CML484	33.15	13.67	Mean	18.23	9.60
CML364 × CML484	31.17	20.82	Group 4: Medium × Medium		
Mean	37.63	17.30	CML396 × CML510	16.30	8.72
Group 3: High × Low			Group 5: Medium × Low		
CML364 × CML254	32.43	15.02	CML381 × CML396	21.29	14.85
CML435 × CML383	26.22	14.98	CML383 × CML396	6.21	3.78
CML364 × CML383	24.22	9.91	CML398 × CML396	8.56	6.20
CML442 × CML383	23.02	5.99	CML451 × CML396	20.19	12.28
CML435 × CML254	19.06	9.36	CML533 × CML396	12.41	9.12
CML364 × CML533	17.83	6.54	CML533 × CML510	11.08	4.90
CML442 × CML254	16.25	3.87	CML381 × CML510	4.00	1.67
CML435 × CML533	14.74	5.85	CML383 × CML510	10.86	7.03
CML442 × CML533	11.03	2.55	CML398 × CML510	14.83	5.62
Mean	20.53	8.23	CML451 × CML510	19.19	6.97
Group 4: Medium × Medium			Mean	12.86	7.24
CML484 × CML376	18.86	9.98			
Group 5: Medium × Low					
CML376 × CML254	11.27	5.81	Group 6: Low × Low		
CML376 × CML383	27.62	5.03	CML381 × CML383	2.00	0.81
CML376 × CML533	7.82	3.28	CML381 × CML398	18.12	10.65
CML484 × CML254	12.97	7.71	CML381 × CML451	16.81	6.47
CML484 × CML383	9.32	6.35	CML381 × CML533	3.03	4.86
CML484 × CML533	12.00	5.36	CML383 × CML398	5.59	3.48
Mean	13.50	5.59	CML383 × CML451	12.26	2.83
Group 6: Low × Low			CML383 × CML533	3.98	0.36
CML254 × CML383	11.57	4.87	CML398 × CML451	7.01	3.12
CML533 × CML254	12.15	4.29	CML398 × CML533	7.27	4.42
CML383 × CML533	13.66	4.90	CML451 × CML533	5.60	3.11
Mean	12.46	4.69	Mean	8.17	4.01
Overall Mean	23.91	10.67	Overall Mean	12.59	6.90

BLUE = Best linear unbiased estimator; HMF = Haploid male fertility; HF: Haploid fertility.

**Table 3:** Mean squares for general combining ability (GCA) and reciprocal, maternal, nonmaternal, and specific combining ability (SCA) and their interactions with environment for maize haploid male fertility (HMF) and haploid fertility (HF).

		Diallel I		Diallel II	
		MS		MS	
Source of variation	df	HMF	HF	HMF	HF
Env	1	1990.28 **	30.60	11.67	22.11
Rep (Env)	2	100.88	46.97	381.11 **	144.90 *
Hybrids	63	1006.67 **	308.48 **	509.47 **	194.94 **
GCA	7	6220.53**	1862.34**	3239.78**	1187.65 **
SCA	28	558.84 **	192.10 **	260.93 **	111.75 **
Reciprocal	28	151.04	36.41	75.42 *	29.94
Maternal	7	155.88	59.73	154.94 **	49.54
No Maternal	21	149.43	28.63	48.92	23.41
Hybrids × Env	63	116.21	29.22	63.42 *	15.10
GCA × Env	7	232.13	37.64	14.84	17.32
SCA × Env	28	111.10	32.47	67.85 *	14.46
Reciprocal × Env	28	92.34	23.87	71.13 *	15.18
Maternal × Env	7	125.83	17.39	226.15 **	38.78
No Maternal × Env	21	81.18	26.03	19.46	7.32
Error	126	142.93	40.85	43.89	43.25

*, ** significant at the 0.01 and 0.05 probability levels, respectively. †ns, nonsignificant at the 0.05 probability level; GCA = general combining ability; SCA = specific combining ability; Env = environment; Rep = replications.

**Table 4:** Estimates of additive and dominance variances, heritability, and ratios of variances of general and specific combining ability for HMF and HF in two diallel experiments.

	Diallel I		Diallel II	
	HMF	HF	HMF	HF
σ^2^_P_	378.44	113.07	169.53	83.12
σ^2^_A_	173.39	52.12	94.85	33.59
σ^2^_D_	62.84	22.41	27.10	13.66
σ^2^_e_	133.68	36.98	43.89	33.87
σ^2^_GCA_\σ^2^_SCA_	1.38	1.16	1.75	1.23
Baker ratio	0.73	0.70	0.78	0.71
h^2^	0.46	0.46	0.56	0.40
H^2^	0.62	0.66	0.72	0.57

HMF = Haploid male fertility; HF = Haploid fertility; σ^2^_P_ = Phenotypic variance; σ^2^_A_ = Additive variance; σ^2^_D_ = Dominance variance; σ^2^_e_ = error variance; H^2^ = broad-sense heritability; h^2^ = narrow-sense heritability; Baker’s ratio = Ratio of additive and dominance variance.

**Table 5:** Estimates of general-combining-ability effects for the parental lines used in two diallels, for HMF and HF.

Diallel I			Diallel II		
Parent	HMF	HF	Parent	HMF	HF
CML254	−10.06 **	−4.58 **	CML364	14.36 **	8.61 **
CML364	13.01 **	8.28 **	CML381	−3.96 **	−1.97 *
CML376	−0.30	−1.45 *	CML383	−7.10 **	−3.77 **
CML383	−9.43 **	−4.59 **	CML396	2.06 *	3.97 **
CML435	9.26 **	6.94 **	CML398	−3.89 **	−2.30 **
CML442	9.48 **	1.22	CML451	−0.15	−1.00
CML484	0.09	0.58	CML510	4.89 **	0.38
CML533	−12.05 **	−6.40 **	CML533	−6.22 **	−3.92 **

**, * significant at the 0.01 and 0.05 probability levels, respectively; HMF = Haploid male fertility; HF = Haploid fertility.

**Table 6:** Estimates of specific combining ability (SCA) effects across two environments in Diallel I and Diallel II.

Diallel I	SCA Effects		Diallel II	SCA Effects	
Hybrids	HMF	HF	Hybrids	HMF	HF
Group1: High × High			Group 1: High × High		
CML364 × CML442	−6.60	−4.58 *	CML364 × CML510	−1.47	1.02
CML435 × CML364	−7.41 *	−3.07	Group 2: High × Medium		
CML435 × CML442	−8.63 *	−3.89 *	CML364 × CML396	−9.11 **	−5.84 **
Group 2: High × Medium			Group 3: High × Low		
CML364 × CML376	−1.88	−5.18 **	CML381 × CML364	−4.97 *	−3.15
CML442 × CML376	−0.57	−2.33	CML383 × CML364	−5.50 **	−2.03
CML435 × CML484	2.09	0.27	CML398 × CML364	−4.11 *	−4.23 *
CML435 × CML376	−2.38	0.47	CML451 × CML364	3.57	2.30
CML442 × CML484	−0.56	0.15	CML533 × CML364	−7.56 **	−6.62 **
CML364 × CML484	−0.89	1.72	Group 4: Medium × Medium		
Group 3: High × Low			CML396 × CML510	−2.58	−2.82
CML364 × CML254	2.28	−0.77	Group 5: Medium × Low		
CML435 × CML383	−1.78	1.92	CML381 × CML396	8.46 **	4.99 **
CML364 × CML383	−7.26	−5.01 **	CML383 × CML396	0.27	−0.58
CML442 × CML383	1.87	1.14	CML398 × CML396	−2.05	−1.81
CML435 × CML254	−1.45	−2.62	CML451 × CML396	3.14	1.92
CML364 × CML533	−5.11	−4.49 *	CML533 × CML396	−0.21	−1.19
CML442 × CML254	−7.81 *	−2.94	CML533 × CML510	1.04	1.26
CML435 × CML533	−5.94	−6.22 **	CML381 × CML510	−4.59 *	−2.24
CML442 × CML533	−8.37 *	−2.00	CML383 × CML510	1.29	1.98
Group 4: Medium × Medium			CML398 × CML510	1.77	0.71
CML484 × CML376	−4.37	−0.49	CML451 × CML510	1.08	0.27
Group 5: Medium × Low			Group 6: Low × Low		
CML376 × CML254	−1.31	2.02	CML381 × CML383	−1.38	−0.54
CML376 × CML383	4.39	−0.56	CML381 × CML398	5.14 **	3.22
CML376 × CML533	−3.43	1.06	CML381 × CML451	1.74	−0.66
CML484 × CML254	−1.75	−0.50	CML381 × CML533	1.32	1.39
CML484 × CML383	−5.92	−2.18	CML383 × CML398	0.70	0.48
CML484 × CML533	2.10	0.33	CML383 × CML451	3.12	0.34
Group 6: Low × Low			CML383 × CML533	1.70	0.50
CML254 × CML383	3.00	1.83	CML398 × CML451	−4.28 *	−1.72
CML533 × CML254	8.05 *	3.85	CML398 × CML533	4.66 *	3.25
CML383 × CML533	9.10 **	3.56	CML451 × CML533	−0.89	0.18

**, * significant at the 0.01 and 0.05 probability levels, respectively. SCA = Specific combining ability; HMF = Haploid male fertility; HF = Haploid fertility.

## Abbreviations

DH	Doubled haploid
HMF	Haploid male fertility
HF	Haploid fertility
GWAS	Genome-wide association study
GP	Genomic prediction
GCA	General combining ability
SCA	Specific combining ability
G×E	Genotype-by-environment interaction
OSR	Overall success rate
